# Contingencies of UTX/KDM6A Action in Urothelial Carcinoma

**DOI:** 10.3390/cancers11040481

**Published:** 2019-04-04

**Authors:** Alexander Lang, Merve Yilmaz, Christiane Hader, Sammy Murday, Xenia Kunz, Nicholas Wagner, Constanze Wiek, Patrick Petzsch, Karl Köhrer, Julian Koch, Michéle J. Hoffmann, Annemarie Greife, Wolfgang A. Schulz

**Affiliations:** 1 Department of Urology, Medical Faculty, Heinrich Heine University, 40225 Düsseldorf, Germany; alexander.lang@hhu.de (A.L.); merve.yilmaz@hhu.de (M.Y.); christiane.hader@hhu.de (C.H.); sammy.murday@klinikumdo.de (S.M.); x.kunz@gmx.de (X.K.); nicholas.wagner@hhu.de (N.W.); michele.hoffmann@hhu.de (M.J.H.); 2 Department of Otolaryngology, Medical Faculty, Heinrich Heine University Düsseldorf, 40225 Düsseldorf, Germany; constanze.wiek@hhu.de; 3 Biological and Medical Research Centre (BMFZ), Heinrich Heine University Düsseldorf, 40225 Düsseldorf, Germany; patrick.petzsch@hhu.de (P.P.); koehrer@hhu.de (K.K.); 4 Chair for Molecular Physical Chemistry, Heinrich Heine University Düsseldorf, 40225 Düsseldorf, Germany; julian.koch@hhu.de (J.K.); annemarie.greife@hhu.de (A.G.)

**Keywords:** bladder cancer, chromatin regulator, histone demethylase, histone methylation, COMPASS complex, RNA-sequencing, nuclear localization, UTX, MLL

## Abstract

The histone demethylase Ubiquitously Transcribed Tetratricopeptide Repeat Protein X-Linked (UTX/KDM6A) demethylates H3K27me2/3 at genes and enhancers and is often inactivated by mutations in urothelial carcinoma (UC). The consequences of its inactivation are however poorly understood. We have investigated the consequences of moderate UTX overexpression across a range of UC cell lines with or without mutations in *KDM6A* or its interaction partners and in a normal control cell line. Effects on cell proliferation, especially long-term, varied dramatically between the cell lines, ranging from deleterious to beneficial. Similarly, effects on global gene expression determined by RNA-Seq were variable with few overlapping up- or downregulated genes between the cell lines. Our data indicate that UTX does not act in a uniform fashion in UC. Rather, its effect depends on several contingencies including, prominently, the status of KMT2C and KMT2D which interact with UTX in the COMPASS complex. In particular, we provide evidence that these factors determine the amount of nuclear UTX.

## 1. Introduction

The histone demethylase Ubiquitously Transcribed Tetratricopeptide Repeat Protein X-Linked (UTX, also known as Lysine demethylase 6A, gene name *KDM6A*) demethylates K27me2/3 on histone 3 (H3), which is usually associated with gene activation [1]. In most instances UTX acts in conjunction with the COMPASS complex [2]. This complex, which mediates H3K4 methylation, comprises a core complex termed WRAD (WDR5, RBBP5, ASH2L and DPY30) and the MLL3 (KMT2C) or MLL2/4 (KMT2D) proteins [3].

UTX is involved in many different human cancers in different ways [1,4]. In some cancer types, UTX exerts pro-tumorigenic effects. For instance, in estrogen receptor-dependent breast cancer UTX serves as a coactivator that facilitates gene activation by the estrogen-receptor α and its co-transcription factors [5]. In other cancer types, UTX function is compromised or abolished by deleterious mutations in the *KDM6A* gene. While the consequences of UTX inactivation are not fully understood, studies on hematological malignancies and pancreatic carcinoma, among others, suggest that it leads to a general increase in H3K27 trimethylation, a redistribution of H3K4 methylation, altered activity of enhancers, and ultimately gene transcription patterns [6,7]. The function of UTX may even differ between subtypes of one disease. For instance, UTX is firmly established as a tumor suppressor in T-ALL, but acts as a protumorigenic coactivator in the TAL1-driven subtype [8,9]. 

Among all cancer types, deleterious *KDM6A* mutations are most frequent in urothelial carcinoma (UC), the most common histological type of urinary bladder cancer. UC is categorized into non-muscle-invasive and more aggressive muscle-invasive tumors, each of which comprise several molecular subtypes [10]. Subtyping of UC is thought to contribute to improved selection of patients for chemotherapy and immunotherapeutic agents. While *KDM6A* mutations are found across all UC stages and subtypes, their frequency varies and is particularly high in non-muscle-invasive low-grade papillary tumors, with up to 70% in female patients [11,12], whereas the frequency is around 25% in muscle-invasive tumors [4]. A few previous publications have addressed the functional consequences of *KDM6A* inactivation in UC, with partly discrepant results. Nickerson et al. [13] observed no differences in monolayer growth of T-24-T cells with a homozygous *KDM6A* nonsense mutation following transfection of a UTX expression plasmid. Likewise, shRNA-mediated downregulation of wild-type UTX in MGH-U3 cells did not affect short-term proliferation. However, colony formation was affected by both treatments. Ahn et al. [14] found an increase in cell proliferation over nine days following *KDM6A* knockout in two UC cell lines (HT-1197 and UM-UC-3). Ler et al. [15] observed a slight increase in proliferation following *KDM6A* knockout in the papillary UC cell line RT-4, whereas restoration of *KDM6A* in the Ku-19-19 cell line, which has a homozygous *KDM6A* mutation, did not appear to affect the basal proliferative activity of this cell line. Interestingly, UC cell lines with constitutive or engineered *KDM6A* inactivation were more sensitive to inhibitors of the H3K27 methyltransferase EZH2, the UTX antagonist [15]. 

In preliminary work, we had observed quite variable responses of UC cell lines to plasmid-based overexpression or knockdown of UTX, especially with respect to long-term clonogenicity. Given the heterogeneous character of UC, we were therefore interested in extending previous observations to further cell lines to map out the heterogeneity of UTX action in this cancer type. To this end, we used a lentiviral vector to transduce UTX fused C-terminally to TagGFP2 or TagGFP2 only into various UC cell lines (UCCs) with different endogenous *KDM6A* status, as well as into a newly engineered UC *KDM6A* knockout cell line and into a non-transformed immortalized urothelial cell line. Among these cell line pairs, we then compared their proliferation rates, clone formation ability, histone modifications and gene expression patterns. We report that responses to UTX expression vary widely among the cell lines and appear strongly contingent on their genetic constitution. In particular, we identify nuclear localization of UTX, which may depend on other COMPASS components, as a new critical point in its action. 

## 2. Results

### 2.1. Generation of Urothelial Cell Lines with Altered UTX Expression

We expressed UTX-TagGFP2 by lentiviral transduction in various UCC and in benign urothelial HBLAK cells (Table 1). For each cell line, a vector control variant was also established. We were primarily interested in the effect of UTX on the cell lines RT112 and VM-CUB-1, which are representative of luminal and basal UC molecular subtypes, respectively [16]. RT112 from a female patient contains a frame-shift mutation in one allele, but expresses UTX from the other one. VM-CUB-1 contains a homozygous, relatively conservative missense mutation (I237N) in one of the UTX TPR repeats. In addition, we investigated 639-V, a cell line wild-type for *KDM6A*, but containing mutations in two important COMPASS genes (*KMT2C* and *KMT2D*), as well as two cell lines lacking functional UTX, namely Ku-19-19, which is homozygous for a *KDM6A* nonsense mutation (Q683*) and T-24, which contains two truncating *KDM6A* mutations (E895*/E902*). As a further control, we used CRISPR/Cas technology to generate a *KDM6A* knockout cell line from SW1710, which is wild-type for KDM6A and related proteins. This knockout cell line was then transduced with UTX-TagGFP2 or TagGFP2.

**Figure 1:**
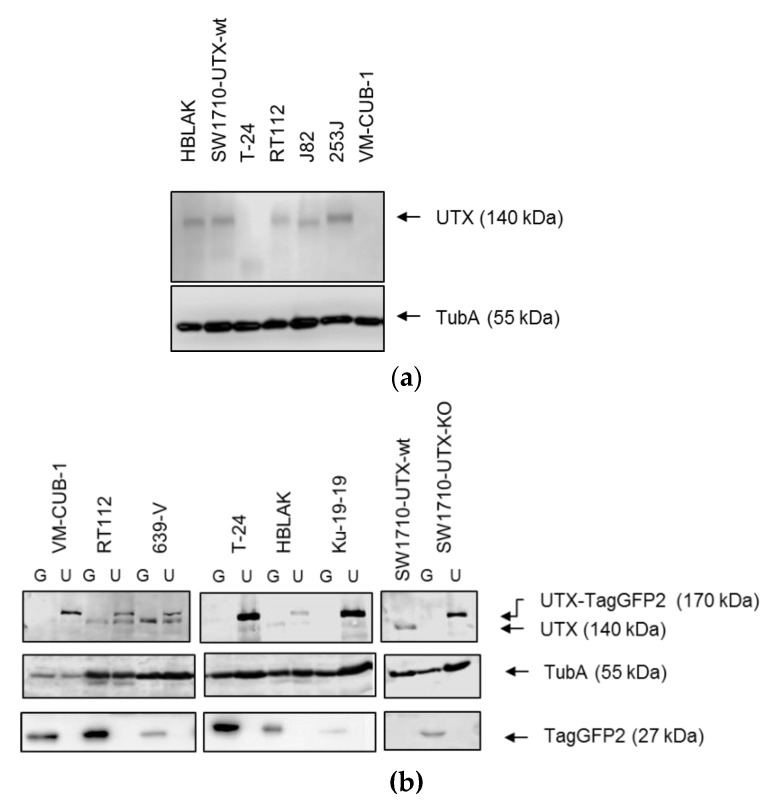
Overexpression of UTX-TagGFP2 in different urothelial (cancer) cell lines. (**a**) Expression of endogenous UTX in various UC cell lines and benign HBLAK urothelial cells. The lower molecular weight band in T-24 could correspond to the truncated proteins expected at ≈100 kDa. (**b**) Detection of UTX-TagGFP2 and endogenous UTX in stably transduced UCCs following selection with Puromycin. G: TagGFP2; U: UTX-TagGFP2. Lower panel: Detection of TagGFP2. α-Tubulin (TubA) was used as a loading control in both panels.

Overexpression and knockout of UTX in all cell variants was confirmed by western blot analysis (Figure 1) and detection of TagGFP2 by fluorescence microscopy (see Section 2.5 below). Notably, levels of UTX were only moderately increased in the successfully transduced cell lines. In all cell lines with at least one wild type copy of KDM6A (RT112, 639-V, HBLAK and SW1710) endogenous UTX could be detected. Interestingly VM-CUB-1, with its homozygous missense mutation, expressed only low levels of the UTX protein. 

### 2.2. Histone Modifications and Chromatin Regulator Proteins in Urothelial Cell Lines with Altered KDM6A Expression

**Figure 2:**
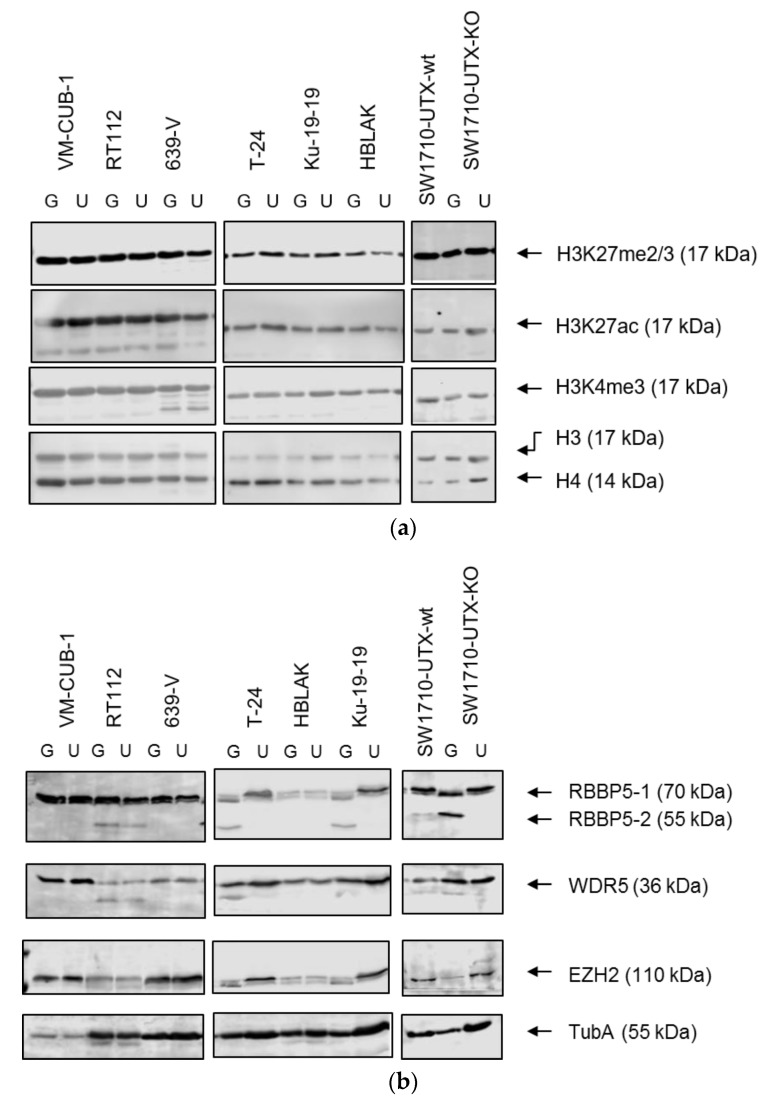
UTX overexpression alters neither global H3 modifications nor protein levels of RBBP5, WDR5, or EZH2 (**a**) Detection of H3 modifications on lysine (K)27 or K4 compared to total H3 and H4 as loading controls (**b**) Detection of RBBP5, appearing as two isoforms, WDR5 and the UTX antagonist EZH2. G: TagGFP2; U: UTX-TagGFP2. α-Tubulin (TubA) was used as loading control in (**b**).

Global cellular levels of relevant histone modifications did not differ substantially between cells transduced with UTX-TagGFP2 or TagGFP2 only. Specifically, the H3 modifications H3K27me2/3, H3K27ac and H3K4me3 were very similar when adjusted to total H3 and H4 levels (Figure 2). 

Likewise, neither overall levels of WDR5, a component of the COMPASS complex nor of EZH2, the H3K27 methyltransferase, were affected by UTX-TagGFP2 expression. Interestingly though, we observed a distinctive effect on the smaller isoform of RBBP5, another core component of the COMPASS complex. Whereas the larger 70 kDa isoform was unaffected, the smaller 55 kDa isoform disappeared upon re-expression of UTX-TagGFP2 in the two cell lines with homozygous *KDM6A* nonsense mutations (T-24 and Ku-19-19) as well as in SW1710 cells following UTX knockout and reintroduction. The mechanism underlying this effect requires further study.

### 2.3. Growth Properties of Urothelial Cell Lines with Altered KDM6A Expression

**Figure 3:**
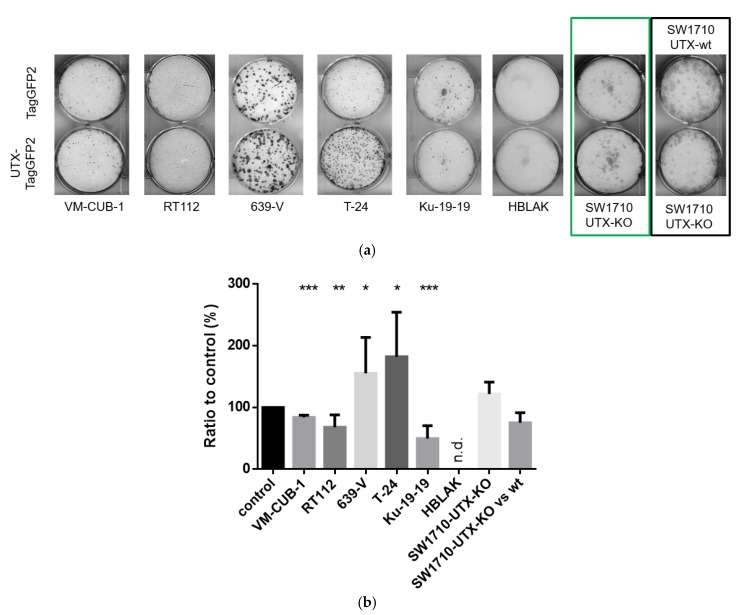

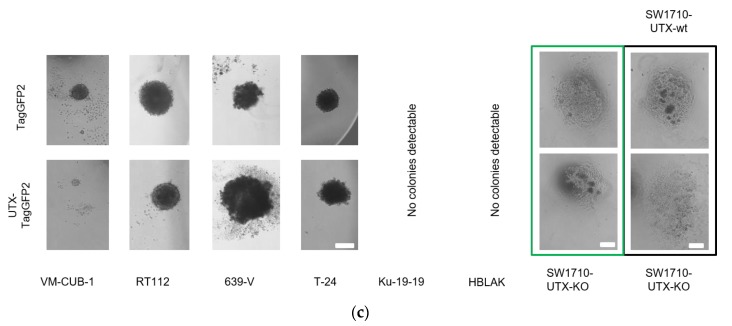
Growth and colony formation assays of UTX-TagGFP2 transduced cell lines. (**a**) Representative examples of 2D clone formation assays in cell lines with TagGFP2 or UTX-TagGFP2; (**b**) Quantitative analysis of these assays; the TagGFP2 control is set as 100% for each cell line; statistics were evaluated by students T-Test (*n* = 3–6; * *p* < 0.05, ** *p* < 0.01, *** *p* < 0.001). (**c**) Representative photographs of colonies in 3D hanging drop cultures. The size and structure of cell colonies from the indicated cell lines were observed using an inverted microscope. Scale: 1 mm. Note that Ku-19-19 and HBLAK cells, with or without transduced UTX, did not grow in this 3D model. Four different variants of SW1710 are shown. The black rectangle frames the comparison of *KDM6A* wildtype and knockout SW1710 cells. The green rectangle frames the comparison of knockout SW1710 cells transduced with TagGFP2 or UTX-TagGFP2. Note that the formation of cell aggregates in SW1710 seems to depend on the presence of UTX.

The cell line pairs transduced with TagGFP2 or UTX-TagGFP2 did not significantly differ from each other in short-term proliferation assays over 3 days (Figure S1). Instead, more pronounced differences were observed in long-term growth assays where cells were seeded at low density (Figure 3). In these assays, UTX-TagGFP2 expressing cells formed significantly fewer colonies in Ku-19-19, RT112 and VM-CUB-1, whereas the number of clones rather increased in 639-V and T-24 cells. *KDM6A* knockout in SW1710 cells rather diminished colony numbers, and this effect was reversed by UTX reintroduction. Notably, while Ku-19-19 and T-24 cells both contain homozygous *KDM6A* nonsense mutations, the two cell lines responded differently to UTX reintroduction. This difference was also evident from the long-term behavior of the two UTX-TagGFP2-transduced cell lines. Whereas the T-24 cells could be grown over longer periods, the UTX-TagGFP2-transduced Ku-19-19 cells could not be propagated over more than 2–3 weeks following several independent transductions.

Striking differences in colony morphology were observed in hanging drop 3D cultures. In keeping with the 2D clone formation assays, RT112 and VM-CUB-1 transduced with UTX-TagGFP2 formed visibly smaller colonies, whereas colonies from 639-V and T-24 cells transduced with UTX-TagGFP2 were substantially or slightly larger, respectively, than those expressing only TagGFP2. The morphology of the colonies also differed. Whereas VM-CUB-1 and RT112 colonies with UTX-TagGFP2 were smoother and tighter compared to the TagGFP2 control, T-24 cells with UTX-TagGFP2 became less compact and formed protrusions. VM-CUB-1 colonies also grew in a more compact pattern in 2D cultures (Figure S2). The gross morphology of 639-V colonies did not change after UTX overexpression.

### 2.4. Gene Expression Changes in Urothelial Cell Lines with Altered KDM6A Expression

Global RNA expression was compared by RNA-Seq between UTX-TagGFP2 and GFP2-only transduced cell line pairs of RT112, VM-CUB-1 and HBLAK. In addition, RNA expression profiles were obtained from SW1710 *KDM6A* knockout cells transduced with UTX-TagGFP2 or TagGFP2 only. Following strict correction for multiple testing by the Bonferroni procedure, 123 genes were significantly differentially expressed between UTX-TagGFP2 and TagGFP2-only transduced RT112 cells (33 up and 90 down in UTX-TagGFP2 cells), 159 genes were differentially expressed between the VM-CUB-1 pair (146 up and 13 down). These numbers remained the same, whether 1.5-fold or 2-fold changes in expression were considered. Between SW1710 *KDM6A* knockout cells transduced with UTX-TagGFP2 or TagGFP2 only 49 genes were differentially expressed (19 up and 30 down) at least 1.5-fold. Intriguingly, no gene retained significant differential expression following adjustment for multiple testing between the HBLAK cell line pairs.

**Figure 4:**
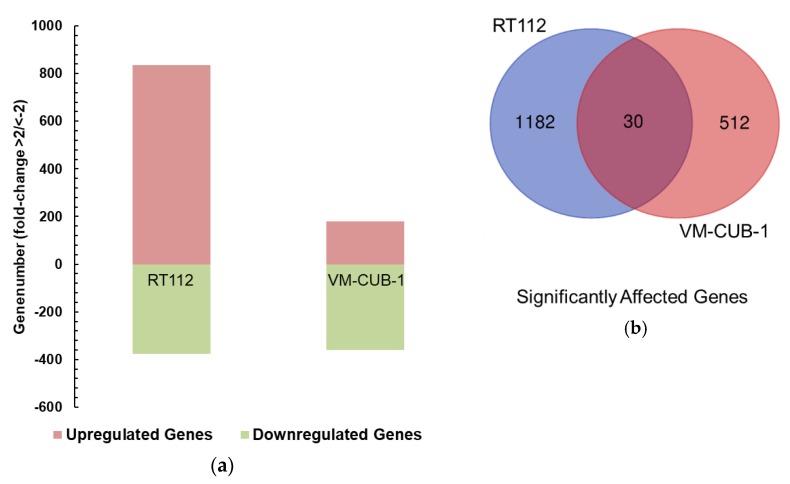
UTX-TagGFP2 modulates gene expression differently between the UCCs VM-CUB-1 and RT112. (**a**) Overview of up- and downregulated genes in VM-CUB-1 and RT112 cell lines upon UTX-TagGFP2 overexpression; (**b**) Venn diagram showing the low overlap between genes (both up and downregulated) differentially regulated in VM-CUB-1 and RT112 cells by UTX-TagGFP2 expression. Genes with *p*(FDR) < 0.05 and > 2-fold change were considered.

**Figure 5:**
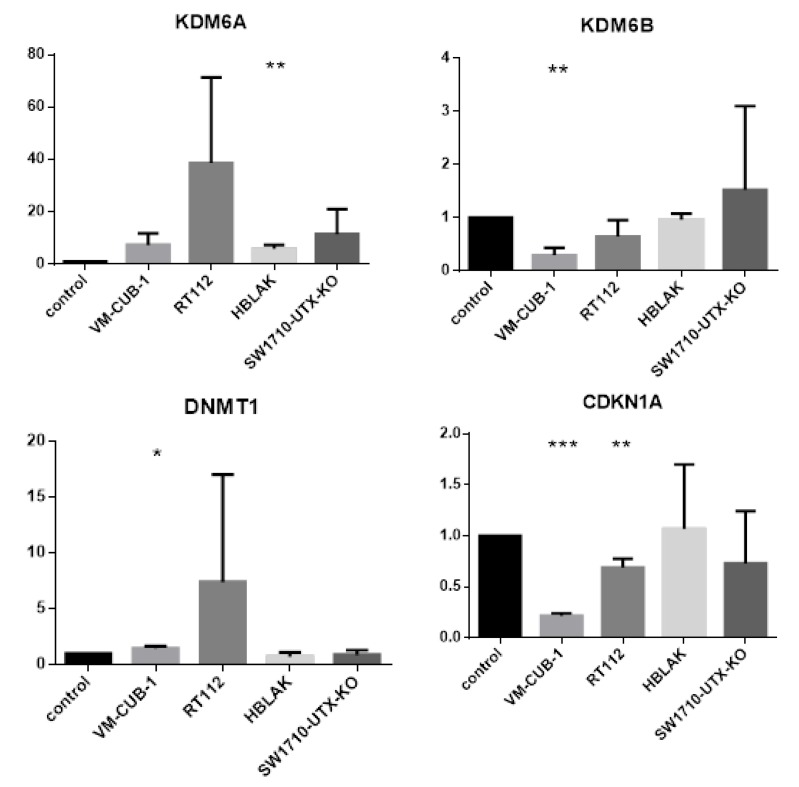
Validation of RNA-seq results by Real-Time-PCR for four representative genes (*DNMT1*, *CDKN1A*/p21, *KDM6B*/JMJD3 and *KDM6A*/UTX). Note that only *KDM6A* itself was regulated in the same direction across the cell lines; differences between UTX-TagGFP2 and TagGFP2 expressing cell lines were evaluated by students *t*-Test (*n* = 3); *p < 0.05, ** *p* < 0.01, *** *p* < 0.001.

After less stringent adjustment by FDR (false discovery rate) the number of differentially expressed genes was substantially raised in the RT112 and VM-CUB-1 cell line pairs to 1212 and 542, respectively (Figure 4). The number of commonly affected genes increased to 30, of which 14 were downregulated and 5 were upregulated in both cell lines, whereas 11 where regulated in opposite directions. While these numbers indicate random overlap, we noted that *DNMT1*, encoding DNA methyltransferase I, was upregulated in both cell lines, and *CDKN1A*, encoding p21^CIP1^, was downregulated in both (Figure 5). Moreover, expression of the paralogous histone demethylase *KDM6B*/JMJD3 was downregulated. In SW1710, use of FDR instead of Bonferroni adjustment increased the number of differentially regulated genes slightly (to 103), but did not change the negative outcome in HBLAK. Few of the differentially regulated genes in SW1710 were also differentially regulated in RT112 and VM-CUB-1 and none was differentially regulated in all three cell lines (see also Figure 5).

**Figure 6:**
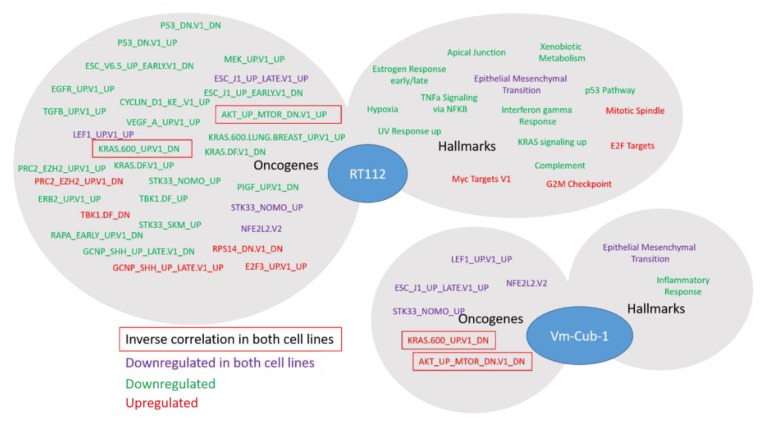
Gene set enrichment analysis (GSEA), performed with the Broad Institute software [15], using the Hallmarks and Oncogenes databases. The analysis is based on the differentially expressed gene sets for RT112 and VM-CUB-1 adjusted for FDR < 5% and 2-fold change.

Even though the individual gene sets affected by UTX overexpression in RT112 and VM-CUB-1 were largely distinct, functional annotation by GSEA (gene set enrichment analysis [17]) of the FDR-adjusted gene set revealed several common themes. Most prominently, genes associated with epithelial-mesenchymal transition (EMT) were downregulated in both cells as well as genes related to NRF2 (*NFE2L2*) response. In contrast, KRAS response-related genes were prominently affected in both cell lines, but in opposite directions. Significant changes in polycomb 2 complex/EZH2-related genes were only seen in RT112 and, interestingly, with both up- and downregulated genes (Figure 6). Indeed, one concordantly regulated gene between the two cell lines was *HOXC4*, which is commonly overexpressed in UC [18]. In RT112, but not in VM-CUB1, further HOX genes were differentially regulated, namely *HOXA1*, *HOXA2*, *HOXA3*, *HOXA13* and *HOXB3*.

GO analysis revealed 35 GO sets shared between RT112 and VM-CUB-1, of which 18 were commonly upregulated and 17 were downregulated (Table S1). The downregulated gene sets included several terms relating to extracellular structure, cell communication, cell membrane composition and adhesion, which reflect the genes identified as related to EMT and KRAS response by GSEA. Interestingly, almost all of the upregulated gene sets were related to RNA biosynthesis and metabolism, including splicing. 

### 2.5. Intracellular Localization of UTX

**Figure 7:**
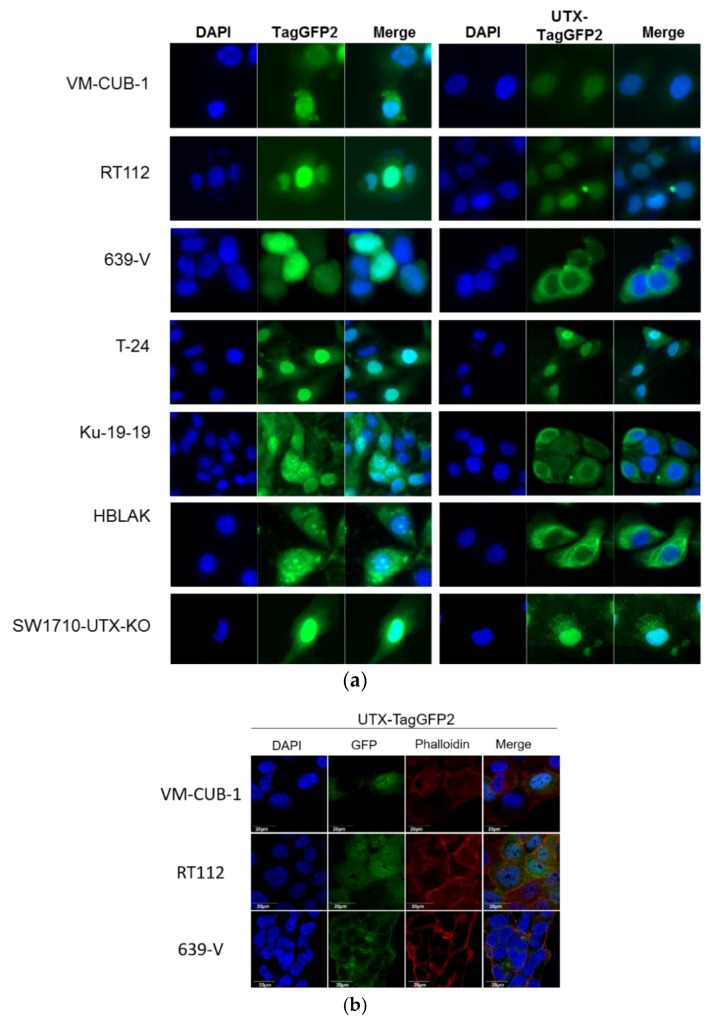
Localization of UTX-TagGFP2 in various cell lines. (**a**) The TagGFP2-signal (green) was detected in cells transduced with TagGFP2 only (left) or UTX-TagGFP2. Scale: 10 µm. (**b**) High-resolution confocal microscopic evaluation of UTX-TagGFP2 expression in selected cell lines. Scale 20 µm. DNA was counterstained with DAPI (blue), F-Actin with Rhodamine Phalloidin. Co-localization is shown in the respective third picture panel (Merge).

Recently, Wiedemuth et al. have raised the issue of the intracellular localization of endogenous and transfected UTX [19]. We therefore investigated the intracellular localization of the introduced UTX-tagGFP2 protein by confocal fluorescence microscopy. Again, the observations indicated considerable differences among the cell lines (Figure 7). Thus, in RT112 and VM-CUB-1 UTX-tagGFP2 was detectable predominantly in the nucleus, while a small fraction of the fusion protein appeared located in the cytoplasm. A similar distribution of UTX-tagGFP2 protein was observed in T-24 and SW1710 cells. In contrast, the protein was almost entirely excluded from the nucleus in transduced 639-V cells. In HBLAK cells, likewise, the protein was largely restricted to the cytoplasm. In Ku-19-19 cells, the UTX-TagGFP2 signal was more prominent in the cytoplasm, but not as exclusively as in 639-V.

### 2.6. Interaction of UTX with the COMPASS Complex

**Figure 8:**
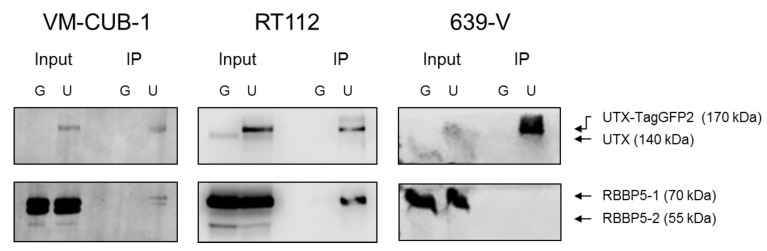
Interaction of UTX-TagGFP2 with RBBP5, a representative component of the WRAD complex in the COMPASS complex, is not detectable in 639-V cells with MLL3/4 nonsense mutations. Following immunopurification with GFP-Trap®_MA beads RBBP5 was detectable with UTX-TagGFP2 in VM-CUB-1 and RT112, but not in 639-V cells. G: TagGFP2; U: UTX-TagGFP2; IP: immunoprecipitation.

The differences in UTX localization among the cell lines raise the question of whether it interacts properly with the COMPASS complex in all UCCs. In particular, whereas VM-CUB-1 and RT112 cells do not contain mutations in *KMT2C*/MLL3 and *KMT2D*/MLL2/4, 639-V cells harbor a homozygous nonsense mutation in *KMT2D* and are heterozygous for a nonsense mutation in *KMT2C*. Since the MLL proteins are difficult to investigate because of their huge size and lack of availability of sufficiently specific antibodies, we investigated whether UTX-TagGFP2 protein interacts with the WRAD component RBBP5. This interaction is thought to be mediated via the MLL proteins [1,2]. Indeed, RBBP5 coprecipitated with UTX-TagGFP2 in VM-CUB-1 and RT112, but not in 639-V cells (Figure 8).

### 2.7. Effect of KMT2C/D Knockdown on Intracellular Localization of UTX

The lack of interaction of UTX-TagGFP2 with COMPASS components could contribute to its mislocalization in 639-V cells. We therefore searched for nuclear localization sequences (NLS) in UTX and other COMPASS complex proteins with the NLS prediction tool NLSdb [20]. Intriguingly, functional NLS were predicted for *KMT2C*/MLL3, *KMT2D*/MLL4, but not for UTX. 

**Figure 9:**
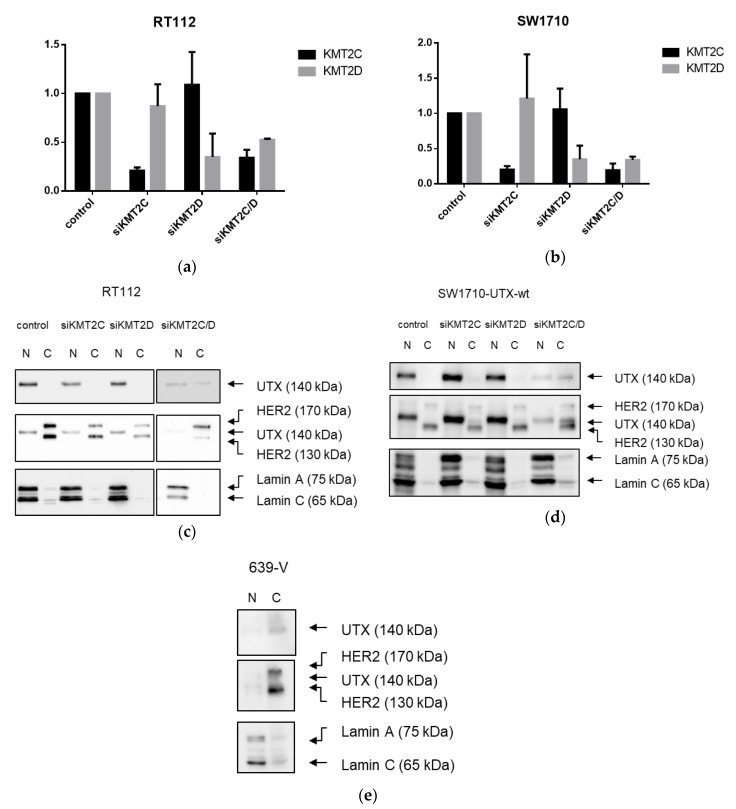
Effects of KMT2C and KMT2D knockdown on UTX intracellular localization. (**a**,**b**) Validation of KMT2C and KMT2D knockdown efficiency in (**a**) RT112 and (**b**) SW1710 cells; (**c**,**d**) Western blot analysis of UTX in nuclear (N) and cytosolic (C) fractions of RT112 and SW1710 cells following knockdown of KMT2C, KMT2D, or both. (**e**) Western blot analysis of UTX in nuclear (N) and cytosolic (C) fractions of 639-V cells. In (**c**–**e**) HER2 and Lamin A/C, respectively, were used as controls for correct fractionation of cytoplasm and nucleus.

Using siRNA against *KMT2C* and *KMT2D* depletion of their mRNA levels to 20–40%, compared to the controls, could be achieved in RT112 and SW-1710 parental cells (Figure 9a,b). As assayed by fractionation into nuclear and cytosolic compartments, UTX remained localized in the nucleus following knockdown of either *KMT2C* or *KMT2D*, as in the cells treated with non-targeting (control) siRNA. In contrast, a substantial fraction of UTX protein remained in the cytoplasm after concomitant knockdown of both genes, *KMT2C* and *KMT2D* (Figure 9c,d). These findings suggest that UTX entry to the nucleus or its retention in this compartment are dependent on the presence of at least one of the MLL proteins. Accordingly, in 639-V cells, which contain a homozygous nonsense mutation in *KMT2D* and a heterozygous nonsense mutation in *KMT2C*, UTX localized much more strongly in the cytoplasm than in the nucleus (Figure 9e).

## 3. Discussion

The high prevalence of evidently deleterious *KDM6A* mutations throughout all stages and molecular subtypes of UC suggests an important tumor-suppressive function of UTX. Unfortunately, the nature of that function has remained rather elusive to date. Concordant with findings of previous publications [13,15], we observed that introduction of UTX did not affect short-term growth of UC cell lines, but in several cell lines impeded long-term proliferation as measured by clone formation assays. These repeated findings suggest that the tumor-suppressive function of UTX in UC is not exerted by fast effects on cell proliferation or survival, as observed during introduction of classical tumor suppressors like p53, but by long-term effects, as one might expect for an epigenetic regulator. We noted however considerable differences between individual UC cell lines in their response to introduced UTX. Obviously, its effects are contingent on several factors in the cell lines. 

First, as one might expect, the effects of exogenous UTX appeared to depend on the status of its endogenous counterpart. Thus, the introduction of functional UTX in cell lines entirely lacking functional protein, such as Ku-19-19, or with hypomorphic genotypes, such as RT112 and VM-CUB-1, effectively diminished clonogenicity. Intriguingly, however, the ability of T-24 cells to form clones was not impaired by UTX. In contrast to this latter finding, Nickerson et al. [13] reported a decreased long-term growth ability of T24-T cells, a T-24 subclone with the same homozygous *KDM6A* nonsense mutation, following transient transfection with a UTX-expression plasmid. One explanation for our finding is that the original T-24 cell line may contain additional genetic and epigenetic changes that allow it to render the action of UTX irrelevant for its long-term growth ability. 

A second contingency is the status of the *KMT2C* and *KMT2D* genes. In UC, mutations in either of these genes are almost mutually exclusive with those in *KDM6A* [4]. This observation indicates that the three proteins, MLL2/4 or MLL3 and UTX, cooperate in the maintenance of a normal state in the urothelium, likely as components of the COMPASS complex. In tumors with *KMT2C* or *KMT2D* inactivation, therefore, the level of UTX expression may be rather irrelevant. This situation is exemplified by the 639-V cell line, which has deleterious mutations in three out of four *KMT2C* and *KMT2D* alleles, but is wild-type for UTX. It is therefore not unexpected that introduction of additional UTX does not impede cell growth in this cell line, or may even promote it.

A novel finding in our study is that exogenous UTX in the 639-V cell line is not localized in the nucleus, but rather in the cytoplasm. In fact, biochemical fractionation demonstrated that the majority of endogenous UTX also localizes to the cytoplasm. Moreover, UTX in this cell line does not co-precipitate with RBBP5, a core component of the COMPASS complex. Taken together, these observations suggest that the COMPASS complex does not properly assemble in 639-V cells and UTX is mislocalized to the cytoplasm. 

Recently, Wiedemuth et al. have drawn attention to the fact that extraneous UTX does not always localize to the nucleus, even in normal cells [19]. We observed this phenomenon, too, in HBLAK cells, a spontaneously immortalized urothelial cell line with a few genomic changes related to immortalization [21], but without mutations in any COMPASS gene. We also note that the UTX protein sequence contains no obvious nuclear localization sequence. Therefore, UTX transport to and retention in the nucleus may depend on further interaction partners. Here, we provide evidence that either of the MLL proteins encoded by KMT2C and KMT2D may serve this function. In particular, knockdown of both factors, combined, but not individually, diminished the fraction of UTX in the nucleus and increased its amount in the cytoplasm. Unfortunately, we could not obtain antibodies specific and sensitive enough to detect endogenous levels of KMT2C and KMT2D proteins in the UCCs. Therefore, we could not evaluate the efficiency of the knockdowns at the protein level. Since the decrease in KMT2C and KMT2D mRNAs was significant, but not complete, it is possible that relevant amounts of these proteins remain after siRNA treatment and further decrease might lead to further exclusion of UTX from the nucleus, as suggested by the observations in the 639-V cell line. 

The findings of Wiedemuth et al. [19] and our own thus hint at the possibility that cells restrict the amount of UTX in their nuclei. Experimentally increased levels of UTX may only saturate the available interaction sites, and additional UTX protein may be retained in the cytoplasm, or become extruded from the nucleus. Taken together, these observations indicate the possibility that transport into the nucleus may constitute another layer of regulation of UTX. 

A limit to the amount of UTX in the nucleus in normal cells could in particular account for the observation that expression of additional UTX did not significantly alter gene expression in HBLAK cells. In contrast, significant effects on gene expression were observed in tumor cell lines with likely suboptimal levels of functional UTX, such as RT112 and VM-CUB-1, and upon reintroduction of UTX into SW-1710 *KDM6A* knockout cells. These changes were observed, even though the relevant global histone modification levels were not appreciably altered, suggesting that they were gene-specific. 

Between the UCCs, the overlap between the differentially expressed gene sets was limited and not larger than expected by chance. Despite this reservation, the intersecting set between RT112 and VM-CUB-1 contained several genes that deserve further investigation as potential UTX target genes. For instance, *DNMT1* encodes the major cellular DNA maintenance methyltransferase and *CDKN1A* encodes the crucial cell cycle inhibitor p21^CIP1^. Inverse regulation of *KDM6A*/UTX and the paralogous histone demethylase *KDM6B*/JMJD3 has been observed in other cancers [22]. Canonical HOX genes are classical, albeit cell type-specific targets of UTX during development [23,24]. Between the two cell lines, only one HOX gene (namely *HOXC4*) was regulated in common by UTX-TagGFP2, whereas others were only affected in RT112 cells.

Whereas the gene expression changes at individual genes were mostly divergent, closer investigation by GSEA revealed several common biological functions. In particular, UTX appeared to negatively regulate genes associated with the epithelial-mesenchymal transition in both RT112 and VM-CUB-1. These changes in gene expression correspond to changes in morphology, namely the more compact colonies observed in 3D and 2D cultures, respectively. Accordingly, Nickerson et al. [13] reported decreased invasion and migration following transfection of UTX into T24-T cells. Differentially expressed KEGG gene sets between *KDM6A*-mutated and wildtype bladder tumors identified by Ler et al. [15] also include prominently ECM_Receptor Interaction, Focal_Adhesion and Cell-Adhesion_Molecules. The possible regulation of cell morphology and adhesion in UC by UTX thus deserves further study. Another interesting observation in our study is that many gene sets, although not individual genes, upregulated by UTX in both RT112 and VM-CUB-1 relate to RNA processing. This may reflect an additional function of UTX during transcription which has been observed in some previous studies [25] but is not well understood in the context of cancer. 

The study by Ler et al. [15] has highlighted the effects of the *KDM6A* status of UC tissues and cell lines on genes repressed by EZH2 and the PRC2 polycomb complex, in keeping with the antagonism between EZH2 and UTX at the biochemical level and during development. In our study, this effect was also apparent in RT112 UTX-TagGFP2 transduced cells, but genes from the PRC2_EZH2 gene set were not uniformly up- or down-regulated. Another developmental gene set was commonly affected in RT112 and VM-CUB-1 cells, namely ESC_J1_UP-LATE.V1_UP; it contains genes upregulated during the late phase of embroid body formation from ESC cells. As for HOX genes, regulation of these genes may reflect the known developmental function of UTX. Finally, in both cell lines, a gene set regulated by the cytoprotective transcription factor NRF2 reacted to UTX-TagGFP2 expression. Generally, relatively little is known about epigenetic regulation of NRF2 action, but, interestingly, one paper reported that the transcription factor was repressed by EZH2 in lung cancer cells [26]; an interaction with UTX has not been reported to the best of our knowledge. 

Importantly, despite some similarities at the level of gene sets, our data do not support the idea that UTX affects a common set of genes across all UC. Gene expression scores for UTX have been suggested based on bioinformatical analyses of NGS data from UC tissues. For instance, like Ler et al. [15], Dancik et al. [27] derived an "UTX activity score" comparing *KDM6A* wildtype and mutant cancers, which differs from that of Ler et al. and consists of much fewer, 27 genes. Only three of these gene were significantly changed in any of our cell line comparisons, namely *USP54* and *TNC* (in RT112) as well as *C1orf21* (in SW-1710 and RT112). While cell lines may not fully reflect regulation of gene expression in vivo, this comparison suggests due caution in the application of such scores, especially in a tumor type like UC which is characterized by high heterogeneity. In particular, Ler et al. [15] have provided evidence that *KDM6A* mutations are predictive for a response of UC to inhibitors of EZH2. In that respect, our findings caution that this response might not be uniform, either.

## 4. Materials and Methods

### 4.1. Cell Lines and Cell Culture

For most experiments, six different UCCs overexpressing TagGFP2 or UTX-TagGFP2 (VM-CUB1, RT112, SW1710, T24, 639-V and KU-19-19) were used. Parental UCCs were obtained from the DSMZ (Braunschweig, Germany) and Dr. H.B. Grossmann (Houston, TX, USA). For comparison, we investigated the spontaneously immortalized normal human urothelial cell line HBLAK (provided by CELLnTEC, Bern, Switzerland) [21]. Cells were cultured and treated in DMEM GlutaMAX-I (Gibco, Darmstadt, Germany) supplemented with 10% fetal calf serum (Biochrom, Berlin, Germany), except for HBLAK cultured in CnT-Prime Epithelial Culture Medium (CELLnTEC, Bern, Switzerland; HBLAK) and KU-19-19 in RPMI-1640 (Gibco), at 37 °C and 5% CO_2_. STR (short tandem repeat) profiling via DNA fingerprint analysis was performed for all cell lines. *KDM6A* and *KMT2C/D* genotypes were obtained from the CCLE database and ascertained by targeted PCR and Sanger sequencing. For individual cell lines, such as HBLAK, whole-exome sequencing data from other own projects [21] was used to elucidate the mutation status. 

### 4.2. Plasmids

*KDM6A* cDNA from the pLenti-C-mGFP-KDM6A plasmid purchased from OriGene (Herford, Germany, RC210861L2) was cloned into the lentiviral vector puc2CL12IPwo using standard techniques, thereby creating the vector puc2CL12IPwo-KDM6A-TagGFP2. Integrity of the *KDM6A* coding sequence was verified by sequencing.

### 4.3. Generation of Cell Lines by Lentiviral Transduction

Lentivirus production and cell transduction was performed as previously described [28,29]. In brief, to produce replication-deficient lentiviruses HEK-293T cells were transfected with helper plasmid expression construct (pCD/NL-BH [30]), envelope vector (pczVSV-G [31]) and the vector plasmids puc2CL12IPwo-TagGFP2 or puc2CL12IPwo-KDM6A-TagGFP2. Viral particles were harvested 48 h after transfection and used to infect UC cells. 24 h after transduction, the supernatant containing viral particles was removed and the transduced cells were selected with 1 µg/mL (2 µg/mL for RT112) puromycin (Invitrogen, Carlsbad, CA, USA) for seven days. Stable overexpression of UTX was confirmed by Western blot analysis of cells from several different passages. Experiments were in general conducted 2–5 passages after transduction. Cells reused after thawing were first retreated with puromycin.

### 4.4. Generation of SW1710 KDM6A Knockout Cells

Cells were transfected using X-tremeGENE 9 DNA Transfection Reagent (Roche, Mannheim, Germany) with KDM6A Double Nickase Plasmid (sc-402761-NIC, SantaCruz Biotechnology, Heidelberg, Germany) encoding a GFP-marker, Puromycin resistance, two different sgRNAs targeting *KDM6A* and Cas9 (Nickase). Transfected cells were selected with 1 µg/mL Puromycin for five days before single-cell seeding into 96-well plates. Genomic DNA was extracted from single-cell clones using QIAamp DNA Mini Kit (Qiagen, Hilden, Germany). An amplicon spanning the sgRNA binding sites was amplified using HotStarTaq polymerase (Qiagen), PCR products were Sanger sequenced. Mutant sequences were compared to the NCBI *KDM6A* reference sequence (NG_016260.1). Successful knockout was verified by western blot analysis for UTX. 

### 4.5. Cell Viability and Clone Formation Assays

Viability of cells was measured after 24 h, 48 h and 72 h of seeding by 3-(4,5-dimetylthiazol-2-yl)-2,5-diphenyltetrazolium bromide dye reduction assay (MTT, M2128-G, Sigma Aldrich, St. Louis, MO, USA).

For colony forming assays, cells were seeded into six-well plates at a density of 500 cells/well. After 8–15 days, cells were fixed in methanol and stained with Giemsa (Merck, Darmstadt, Germany). Colony forming units were counted by Fiji (1.52b) with the BioVoxxel Toolbox [32] using the green channel of a 300 dpi RGB picture. One well of a six-well plate was sectioned (makeOval) and a filter ("Mean...", "radius = 2") was used. Via Threshold (Triangle) a binary picture was generated and fused colonies were separated (Watershed). Binary particles were measured by the Analyze Particles tool. 

For hanging-drop culture cells were seeded into the lids of six-well plates at a density of 5000 cells/drop in a volume of 20 µL of culture media. The wells were filled with sterile phosphate buffered saline (PBS) to prevent desiccation. After 4 days colonies in the drops were photographed using an inverted microscope.

### 4.6. Western Blot Analysis

Total protein extraction, purification of histones and Western blot analysis were performed as previously described [33]. Briefly, cells were incubated for 30 minutes on ice in RIPA-buffer (150 mM NaCl, 1% Triton X-100, 0.5% desoxycholate, 1% Nonidet P-40, 0.1% SDS (sodium dodecyl sulfate), 1 mM EDTA, 50 mM TRIS (pH 7.6)) containing 10 µL/mL protease inhibitor cocktail (#P-8340, Sigma Aldrich). Histones were extracted by a modified published protocol employing sulphuric acid extraction and TCA-precipitation [34]. Concentrations of total protein and histones were determined by BCA protein assay (Thermo Fisher Scientific, Carlsbad, CA, USA). Subsequently, total cell proteins (50 µg) or extracted histones (2 µg) were separated by SDS-PAGE (total proteins 10% gels, histones 15% gels), transferred to PVDF membranes (Merck Millipore, Berlin, Germany) and were incubated with primary antibodies at RT for 1 h or 4°C overnight (see Table S2) following blocking with 5% non-fat milk in TBST (150 mM NaCl, 10 mM TRIS, pH 7.4 and 0.1% Tween-20). For signal detection membranes were incubated with a suitable horseradish peroxidase-conjugated secondary antibody (see Table S1) at RT for 1 h and signals were visualized by Clarity™ Western ECL Substrate (Bio-Rad Laboratories, Munich, Germany) and WesternBright Quantum kit (Biozym, Hessisch Oldendorf, Germany).

### 4.7. RNA Extraction and Reverse Transcription

Total cell RNA was isolated by the Qiagen RNeasy Mini Kit (Qiagen) according to the manufacturer’s protocol and cDNA was synthesized using QuantiTect Reverse Transcription Kit (Qiagen) with an extended incubation time of 30 min at 42 °C as previously described [33]. Target mRNA expression was measured by qRT-PCR with QuantiTect SYBR Green RT-PCR Kit (Qiagen) on the LightCycler® 96 Real-Time PCR system with software version 1.1 (Roche Diagnostics, Rotkreuz, Switzerland). All used primers, comprising QuantiTect Primer assays (Qiagen), self-designed target primers and primers for the reference housekeeping gene *TBP* (TATA-box binding protein), are listed in Table S2.

### 4.8. RNA-Seq and Data Analysis

Total RNA samples used for transcriptome analyses were quantified (Qubit RNA HS Assay, Thermo Fisher Scientific) and quality measured by capillary electrophoresis using the Fragment Analyzer and the Total RNA Standard Sensitivity Assay (Agilent Technologies, Inc. Santa Clara, CA, USA). All samples in this study showed high quality RNA Quality Numbers (RQN; mean = 9.9). The library preparation was performed according to the manufacturer’s protocol using the ‘VAHTS™ Stranded mRNA-Seq Library Prep Kit’ for Illumina®. Briefly, 300 ng total RNA were used for mRNA capturing, fragmentation, the synthesis of cDNA, adapter ligation and library amplification. Bead purified libraries were normalized and finally sequenced on the HiSeq 3000/4000 system (Illumina Inc. San Diego, CA, USA) with a read setup of 1 × 150 bp. The bcl2fastq tool was used to convert the bcl files to fastq files as well for adapter trimming and demultiplexing. 

Data analyses on fastq files were conducted with CLC Genomics Workbench (version 10.1.1, QIAGEN, Venlo, The Netherlands). The reads of all probes were adapter trimmed (Illumina TruSeq) and quality trimmed (using the default parameters: bases below Q13 were trimmed from the end of the reads, ambiguous nucleotides maximal 2). Mapping was done against the Homo sapiens (hg38) (Mai 25, 2017) genome sequence. After grouping of samples (three biological replicates each) according to their respective experimental condition, multi-group comparisons were made and statistically determined using the Empirical Analysis of DGE (version 1.1, cutoff = 5). The Resulting *p* values were corrected for multiple testing by FDR and Bonferroni-correction. A *p* value of ≤ 0.05 was considered significant.

### 4.9. Gene Set Enrichment Analysis (GSEA)

GSEA [18] was performed with the software provided by https://software.broadinstitute.org/gsea/index.jsp, and performed with the MSigDB database v6.2. The datasets generated by RNA-Seq were split into two based on up- and downregulated genes upon UTX-TagGFP2 overexpression compared to TagGFP2 overexpression and were statistically significant for multiple testing by FDR. Each dataset was used to correlate the gene set to GSEA Hallmarks, Oncogenes and Gene Ontology (GO) gene sets. A group was considered upon an enrichment of at least 15 genes and a statistical significance of FDR < 25%. 

### 4.10. Immunocytochemistry

Analysis of UTX-TagGFP2 localization was performed in stably transduced UCCs. As previously described [33], after fixation with 4% formaldehyde, cells were permeabilized using 0.2% Triton X100 in PBS for 10 min at RT, blocked with 1% BSA in PBS, for 30 min at RT and subsequently incubated for 1 h at RT with 14 nM Rhodamine Phalloidin in blocking solution. Following counter-staining of nuclei with 1 µg/mL DAPI (4´,6-diamidino-2-phenylindole) cells were mounted with fluorescence mounting medium (DAKO, Glostrup, Denmark). Imaging was performed using ZEISS Axio Observer.Z1 / 7; Plan-Apochromat 40x/1.4 Oil DIC (UV) VIS-IR M27; 90 HE DAPI/ GFP/ Cy3 /Cy5; LED-module "wavelength" nm (Colibri 7); Axiocam 512 mono (ZEISS, Jena, Germany)

Higher resolution images were obtained using an inverted confocal FluoView1000 laser scanning microscope with a 60xW UPLSAPO objective NA1.2 in a sequential DAPI-GFP-Phalloidin scanning mode (Olympus, Hamburg, Germany). Resolution was 1024 × 1024. 

### 4.11. Immunopurification

Cells were harvested at a cell count of ≈1 × 10^7^ cells and lysed as described in Section 4.6. The immunopurification was performed with 2 mg cleared cell lysate, from which an aliquot was used as input control, and 10 µL magnetic GFP-beads (GFP-Trap®_MA ChromoTek, Martinsried, Germany) of 50% slurry (washed three times in lysis buffer) overnight at 4 °C under permanent rotation. Then, beads were washed three times with lysis buffer, incubated with 1× Laemmli denaturing solution and heated at 95 °C for 5 min. The entire beads fraction and 50 µg input protein each were loaded on a Tris-Glycin-SDS-PAGE. Subsequent steps were performed as described in Section 4.6.

### 4.12. Transient siRNA Transfection

For transient transfection of siRNA the Lipofectamine™ RNAiMAX Transfection Reagent (13778030, Invitrogen) was used. Cells were seeded at a number of 2 × 10^5^ (RT112) or 1.5 × 10^5^ (SW1710) per well of 6-well plates and cultured for one day at 37 °C and 5% CO_2_. Then, cells were transfected with 4 nM of siRNA as described in the RNAiMAX datasheet. Media were changed one day after transfection. Cells were harvested after 3 days and RNA and proteins were isolated as described above or below, respectively.

### 4.13. Nuclear and Cytoplasmic Fractionation

Fractionation of cytoplasmatic and nuclear proteins was performed using three buffers: HB-Buffer (10 mM Tris-HCl, pH 8, 10 mM KCl, 1.5 mM MgCl_2_, 0.5 mM 2-mercaptoethanol, protease inhibitor [P8340, Merck; 4 µL per 5 mL]); Lysis-Buffer: HB-Buffer + 0.4% NP-40); Buffer C (20 mM HEPES, 400 mM NaCl, 1 mM EDTA, 1 mM DTT, protease inhibitor [P8340, Merck; 4 µL per 1 mL]). 1–5 × 10^6^ cells were harvested, pelleted at 200× *g* for 5 min and washed at RT. The cell pellet was resuspended in 350 µL HB-Buffer and centrifuged at 200× *g* for 1 min at 4 °C. The supernatant was removed and the pellet was resuspended in 100 µL Lysis-Buffer followed by incubation for 15 min at 4 °C. The supernatant containing the cytosolic fraction was harvested by centrifugation at 15,000× *g* for 5 min at 4 °C. The pellet was washed twice with 75 µL Lysis-Buffer, resuspended in 60 µL Buffer C and incubated for 15 min at 4 °C. Following centrifugation at 15,000× *g* for 5 min at 4 °C the supernatant containing the nuclear fraction was harvested. Protein concentrations were then determined using the Protein Assay Kit I (Bio Rad, 5000001).

### 4.14. Statistical Methods

*p*-values between different groups were determined by the Student´s *t*-test; asterisks denote significant (* < 0.05) differences; error bars indicate SD. The RNA-Seq data is available from the authors upon reasonable request.

## 5. Conclusions

Although mutations of the epigenetic regulator UTX/*KDM6A* occur at a similar frequency as those in p53 in urothelial carcinoma, their consequences are poorly understood. Here, we show that introduction of functional UTX/*KDM6A* has quite diverse effects in urothelial carcinoma cell lines representing a broad spectrum of genotypes and phenotypes that reflect the heterogeneity of this cancer type. In cell lines with *KDM6A* deficiencies, introduction of functional UTX generally diminished long-term, but not short-term growth. Despite this similar overall effect, gene expression changes elicited by the epigenetic regulator showed little overlap among the cell lines; however, regulation of EMT may constitute one commonly affected pathway.

The causes for this diversity appear to reside in several contingencies, primarily the endogenous status of *KDM6A*, but also of its interacting MLL proteins encoded by *KMT2C* and *KMT2D*. In particular, our findings suggest that these proteins may determine the amount of UTX protein in the nucleus and that even in normal cells this amount may be restrained. Our study thus suggests a new level of regulation of UTX activity and moreover, that the activity of wild-type UTX may be substantially compromised by mutations in *KMT2C* and *KMT2D* in UC and likely other cancers. 

## Figures and Tables

**Table 1:** Properties of cell lines used.

Cell Line	Tumor Information	Sex	AA Changes		
			*KDM6A*	*KMT2C*	*KMT2D*
VM-CUB-1	UC	male	I1237N	wt	Q3815*
				wt	Q2863Q
RT112	UC, G2 papillary	female	P1139fs*19	A1691T	wt
			wt	wt	wt
639-V	UC, G3	male	wt	R2028*	R1429*
				wt	R1429*
T-24	UC, G3	female	E895*	wt	wt
			E902*	wt	wt
Ku-19-19	UC, G3	male	Q863*	wt	T2171fs*44
				wt	wt
SW1710	UC, G3 papillary	female	wt	wt	wt
			wt	wt	wt
HBLAK	Normal urothelium	male	wt	wt	wt
				wt	wt

*: nonsense (truncating) mutation.

## Supplementary Materials

The following are available online at https://www.mdpi.com/2072-6694/11/4/481/s1, Table S1: GO-pathways commonly regulated in RT112 and VM-CUB-1 by UTX-TagGFP2, Table S2: Antibodies for western blot analysis, Figure S1: Urothelial cancer cells transduced with UTX-TagGFP2 show no significant changes in cellular vitality measured by MTT assay over a period of three days, Figure S2: Morphology of urothelial cancer cell lines transduced with UTX-TagGFP2. Note the more compact growth pattern of colonies especially in VM-CUB-1 cells, Table S3: Primers used for quantification of selected genes by Real-Time-PCR, Table S4: Used siRNAs for specific gene expression knockdown.
