# Supplementary material for: Contingencies of UTX/KDM6A Action in Urothelial Carcinoma
Source: Cancers (Basel). 2019 Apr 4;11(4):481. doi: 10.3390/cancers11040481 (PMC6520694; doi:10.3390/cancers11040481)
Supplement: Supplementary file 1 [file cancers-11-00481-s001.pdf]

Supplementary Materials: Contingencies of UTX/KDM6A action in urothelial carcinoma

Alexander Lang, Merve Yilmaz, Christiane Hader, Sammy Murday, Xenia Kunz, Nicholas Wagner, Constanze Wiek, Patrick Petzsch, Karl Köhrer, Julian Koch, Michéle J. Hoffmann, Annemarie Greife and Wolfgang A. Schulz

Table S1. GO-pathways commonly regulated in RT112 and VM-CUB-1 by UTX-TagGFP2.

Upregulated GO-Pathways	Downregulated GO-Pathways
GO_MRNA_METABOLIC_PROCESS	GO_SECRETORY_VESICLE
GO_RIBONUCLEOPROTEIN_COMPLEX_BIOGENESIS	GO_EXTRACELLULAR_SPACE
GO_NCRNA_METABOLIC_PROCESS	GO_REGULATION_OF_RESPONSE_TO_EXTERNAL_STIMULUS
GO_RIBONUCLEOPROTEIN_COMPLEX	GO_REGULATION_OF_ANATOMICAL_STRUCTURE_MORPHOGENESIS
GO_ATPASE_ACTIVITY	GO_EXTRACELLULAR_STRUCTURE_ORGANIZATION
GO_POLY_A_RNA_BINDING	GO_POSITIVE_REGULATION_OF_CELL_PROLIFERATION
GO_RNA_BINDING	GO_RESPONSE_TO_OXYGEN_CONTAINING_COMPOUND
GO_NUCLEOPLASM_PART	GO_CATION_TRANSMEMBRANE_TRANSPORTER_ACTIVITY
GO_POSTTRANSCRIPTIONAL_REGULATION_OF_GENE_EXPRESSION	GO_POSITIVE_REGULATION_OF_CELL_COMMUNICATION
GO_RNA_SPLICING	GO_PROTEINACEOUS_EXTRACELLULAR_MATRIX
GO_RNA_SPLICING_VIA_TRANSESTERIFICATION_REACTIONS	GO_POSITIVE_REGULATION_OF_RESPONSE_TO_STIMULUS
GO_NUCLEOLUS	GO_INTRINSIC_COMPONENT_OF_PLASMA_MEMBRANE
GO_NCRNA_PROCESSING	GO_REGULATION_OF_ION_TRANSPORT
GO_CHROMOSOME_ORGANIZATION	GO_BIOLOGICAL_ADHESION
GO_MRNA_PROCESSING	GO_RESPONSE_TO_CYTOKINE
GO_RNA_PROCESSING	GO_POSITIVE_REGULATION_OF_INTRACELLULAR_SIGNAL_TRANSDUCTION
GO_CHROMOSOME	GO_ENDOPLASMIC_RETICULUM
GO_DNA_METABOLIC_PROCESS	

Table S2. Antibodies for western blot analysis.

Antibody	Supplier	ID	Dilution
UTX	Cell Signaling Technology	# 33510	1:500
RBBP5	Cell Signaling Technology	# 13171	1:1.000
WDR5	Cell Signaling Technology	#13105	1:1.000
EZH2	Cell Signaling Technology	#3147	1:1.000
TubA	Abcam	ab4074	1:50.000
GFP	Cell Signaling Technology	#2955	1:1.000
H3	Cell Signaling Technology	#4499	1:1.000
H4	Active Motif	#61199	1:1.000
H3K27me2/3	Active Motif	#39535	1:1.000
H3K27ac	Active Motif	#39133	1:1.000
H4K4me3	Active Motif	#39915	1:1.000
Lamin A/C	Cell Signaling Technology	#2032	1:1.000

HER2	Cell Signaling Technology	#2165	1:1.000
Rabbit Anti-Mouse Immunoglobulins/HRP	Dako	P0260	1:2000
Goat Anti-Rabbit Immunoglobulins/HRP	Dako	P0448	1:2000

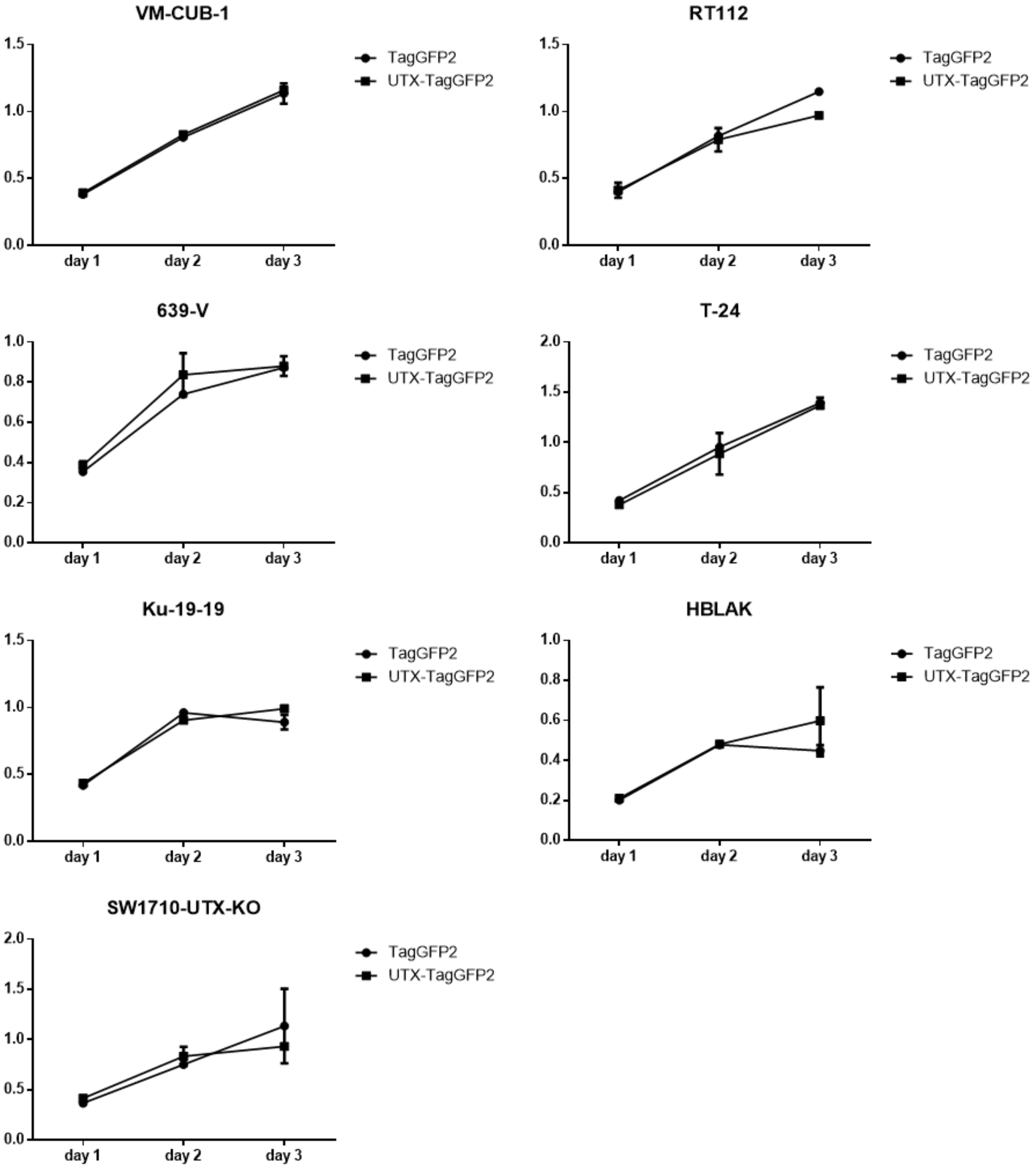

Figure S1. Urothelial cancer cells transduced with UTX-TagGFP2 show no significant changes in cellular vitality measured by MTT assay over a period of three days.

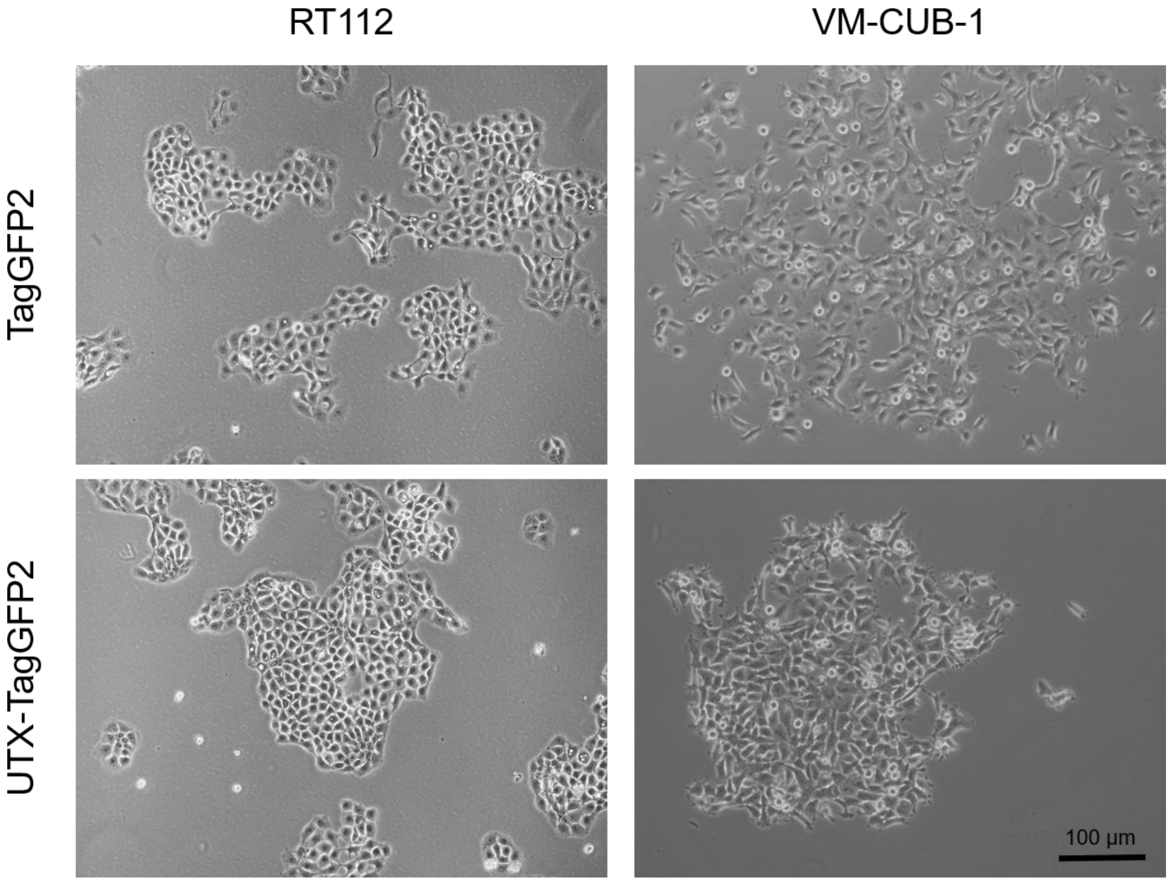

Figure S2. Morphology of urothelial cancer cell lines transduced with UTX-TagGFP2. Note the more compact growth pattern of colonies especially in VM-CUB-1 cells. .

Table S3. Primers used for quantification of selected genes by Real-Time-PCR.

Gene	Fw (5′->3′):	Rev (5′->3′):	Annealing Temperature
KDM6A	GGCGATAAAGTTGGTGTGCT	GAACAGCCTTGCCCAGTAG	55 °C
KDM6B	GACCCCGACTTGTTCAAGA	CCCTGGTAAGCGATTTTCT	55 °C
CDKN1A	GGAAGACCATGTGGACCTGT	GGCGTTTGGAGTGGTAGAAA	55 °C
DNMT1	GGCTATCAGTGCACCTTCG	GGAGCAAACACGTGCAGTG	55 °C
TBP	ACAACAGCCTGCCACCTTA	GAATAGGCTGTGGGGTCAGT	55 °C
KMT2C	TTACACACAGTGCGCTCCTT	AGGGTCTGCACATGCTACAA	55 °C
KMT2D	CGCAGTGCTCTCAGTGCTAT	AGTCATCACAGAGCAGCAGG	55 °C

Table S4. Used siRNAs for specific gene expression knockdown.

siRNA	Supplier	ID	Used concentration
KMT2C	Dharmacon	L-007039-00-0005	8 nM (or 4 nM in double transfection)
KMT2D	Dharmacon	L-004828-00-0005	8 nM (or 4 nM for double transfection)
control	Dharmacon	D-001810-01-05	8 nM
